# Successful Early Treatment of Anti‐N‐Methyl‐d‐Aspartate Receptor Encephalitis Associated With Small Cell Lung Cancer in an Elderly Male Patient: A Case Report

**DOI:** 10.1002/npr2.70044

**Published:** 2025-08-26

**Authors:** Kohei Kamikawa, Ryohei Takada, Yuya Honda, Harue Goto, Takashi Okada

**Affiliations:** ^1^ Department of Psychiatry Nara Prefecture General Medical Centre Nara Japan; ^2^ Department of Psychiatry Nara Medical University School of Medicine Kashihara Japan

**Keywords:** anti‐N‐methyl‐d‐aspartate receptor (NMDAR) encephalitis, immunotherapy, late onset, limbic encephalitis, small cell lung carcinoma (SCLC)

## Abstract

**Background:**

Anti‐N‐methyl‐d‐aspartate receptor (NMDAR) encephalitis, a type of autoimmune encephalitis, characterized by acute onset neuropsychiatric symptoms, predominantly affects young females and is often associated with ovarian teratomas. Although small cell lung cancer (SCLC) is a known cause of paraneoplastic encephalitis, its association with anti‐NMDAR encephalitis is rare and often carries a poor prognosis due to limited response to immunotherapy.

**Case Presentation:**

An 80‐year‐old male with no psychiatric history presented with flu‐like symptoms, followed by the acute onset of neuropsychiatric symptoms, including pressured speech, agitation, memory impairment, and abnormal behavior. Autoimmune encephalitis was suspected due to mildly elevated cerebrospinal fluid (CSF) white cell count and a mass in the right upper lung detected by whole‐body computed tomography (CT) on the first day of hospitalization. High‐dose intravenous corticosteroids were administered on Day 1, resulting in prompt and sustained improvement in symptoms. CSF was later confirmed positive for anti‐NMDAR antibodies, and a bronchoscopy biopsy of the pulmonary mass diagnosed SCLC. The patient recovered without neurological deficits and was discharged in stable condition on hospital Day 30.

**Conclusion:**

This was a rare case of anti‐NMDAR encephalitis associated with SCLC in an elderly male patient. Diagnosis in elderly individuals is often challenging because of the atypical presentations and lower tumor association. Nevertheless, timely intervention initiation may lead to favorable outcomes. Clinicians should consider autoimmune encephalitis, including anti‐NMDAR encephalitis, when evaluating acute onset neuropsychiatric symptoms in elderly individuals and initiate early immunotherapy alongside tumor screening.

## Abstract 2

An 80‐year‐old male developed acute neuropsychiatric symptoms and was diagnosed with anti‐NMDAR encephalitis associated with small cell lung cancer. Early corticosteroid therapy led to full recovery. This case emphasizes the importance of early diagnosis and prompt immunotherapy, even in elderly patients, for favorable outcomes in autoimmune encephalitis.

## Graphical abstract

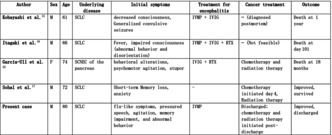

## 1 Background

Anti‐N‐methyl‐d‐aspartate receptor (NMDAR) encephalitis is an autoimmune encephalitis first reported by Dalmau et al. [1], with autoantibodies against NMDAR (particularly the NR1 subunit), a critical component of synaptic transmission and plasticity, implicated as the etiological factor [2, 3, 4, 5, 6]. This disease is strongly associated with paraneoplastic encephalitis, predominantly affecting young females; approximately 50% of female patients present with ovarian teratomas [5, 7]. A large observational study by Dalmau et al. showed a wide age range of 8 months to 85 years, with a median age of 21 years, and 81% female, indicating that the disease predominantly affects young females [7]. Tumor complications were observed in 38% of patients, of which 97% were in females [7]. Among females, 46% had tumors, with ovarian teratomas accounting for 94% of these cases [7]. In contrast, only 5% of males developed tumor‐related complications, primarily testicular germ cell tumors and small cell lung cancer (SCLC) [7]. A separate study found that tumor association was present in 23% of patients aged over 45 years compared to 51% in those under 45 years of age, indicating that tumor‐related complications are less frequent in older individuals [7]. In elderly patients, tumors of the lungs, breasts, testes, ovaries, uterus, thymus, and pancreas have also been reported, replacing ovarian teratomas with the predominant tumor type [7, 8]. Clinically, anti‐NMDAR encephalitis typically follows a multiphasic course, beginning with flu‐like symptoms and progressing to the acute onset of neuropsychiatric symptoms such as impaired consciousness, seizures, involuntary movements, and autonomic dysfunction [5, 7, 9]. On the other hand, typical seizures and involuntary movements (such as oral‐lingual dyskinesia) are less frequently observed in elderly individuals than in younger individuals, which may delay diagnosis and treatment [10, 11, 12]. Moreover, a poor response to immunotherapy has been linked to worse prognoses [13]. SCLC is a common malignancy in elderly individuals and a well‐known cause of paraneoplastic neurological syndrome (PNS). However, its association with anti‐NMDAR encephalitis is rare [14]. The few cases reported to date have typically been unresponsive to immunotherapy and have shown poor prognosis [15, 16]. Herein, we report the case of an elderly male with anti‐NMDAR encephalitis associated with SCLC who achieved a favorable outcome through early diagnosis and treatment.

## 2 Case Presentation

**FIGURE 1:**
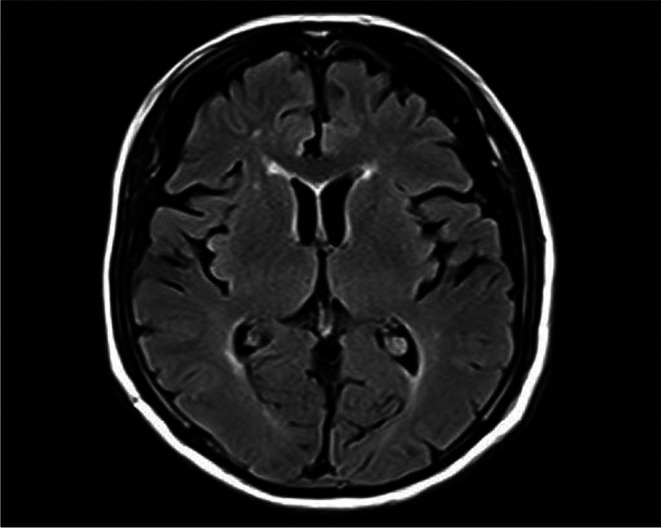
FLAIR‐weighted brain MRI on hospital day 1 showing no localized atrophy or abnormal signal intensity. No encephalitic changes were observed. FLAIR, Fluid‐attenuated inversion recovery; MRI, Magnetic resonance imaging.

**FIGURE 2:**
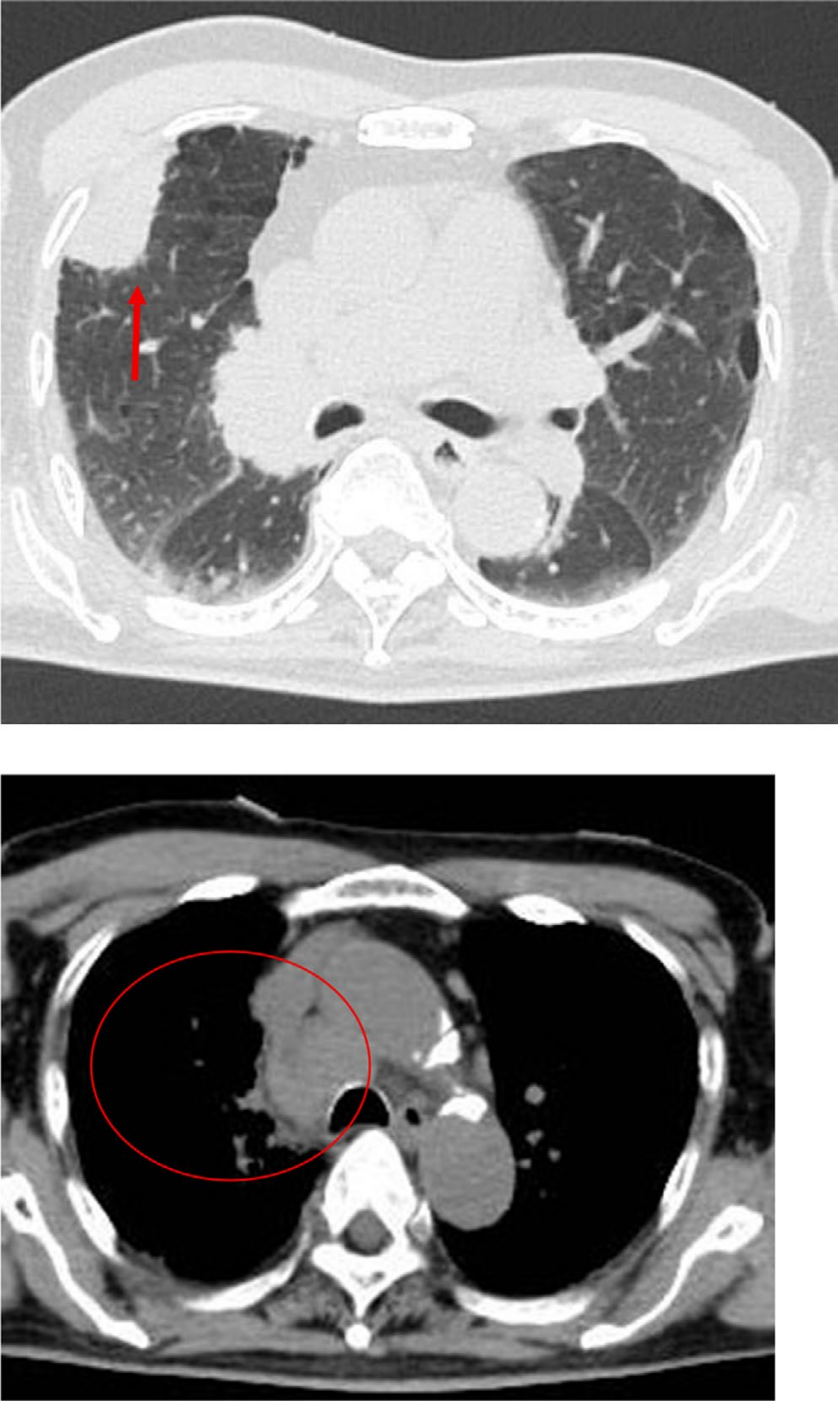
Whole‐body CT on hospital day 1. The red arrow indicates a mass in the right upper lung field. The red circle outlines the area of lymphadenopathy in the mediastinum and right hilar region. The upper and lower images were reconstructed under the lung and mediastinal window settings, respectively. CT, Computed tomography.

**FIGURE 3:**
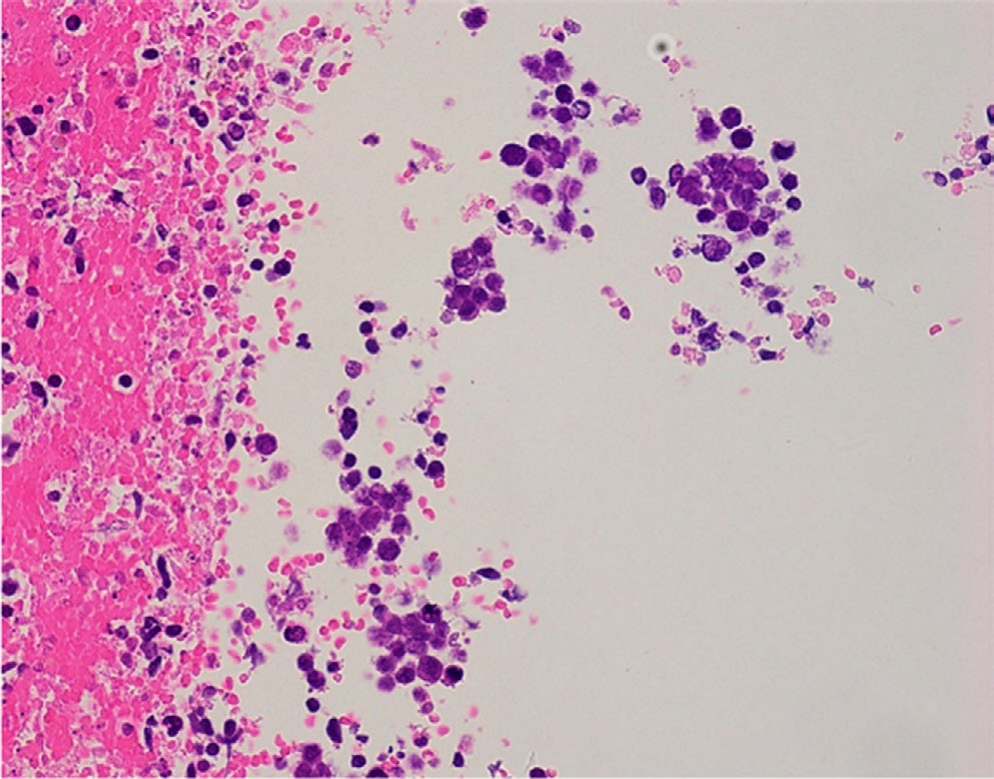
Hematoxylin and eosin staining reveals neoplastic cells with a high nuclear‐to‐cytoplasmic ratio and evidence of nuclear division (original magnification ×40).

An 80‐year‐old male had no history of psychiatric disorder. He exhibited no memory impairment and was fully independent in activities of daily living (ADLs) prior to symptom onset. Approximately 10 days before hospitalization, the patient presented with flu‐like symptoms including fever, headache, and fatigue. Approximately 3 days before hospitalization, he began to show pressured speech, agitation, and short‐term memory impairment. He also exhibited abnormal behaviors such as loud shouting and moving his hands without an apparent reason. Due to the rapid progression of these symptoms, dementia or primary psychiatric disorders were suspected, and the patient was admitted to our psychiatric department at the Nara Prefecture General Medical Center. Neurological examination on the first day of hospitalization (hospital day 1) revealed mild nuchal rigidity. No abnormalities were noted in cranial nerve function, motor strength, deep tendon reflexes, cerebellar coordination, or sensory modalities. The patient also exhibited impaired orientation and short‐term memory, which were interpreted as signs of mild disturbance of consciousness. Hospital day 1, fluid‐attenuated inversion recovery (FLAIR)‐weighted brain magnetic resonance imaging (MRI) revealed no localized atrophy or abnormal signal intensity in the medial temporal lobe (Figure 1). However, whole‐body computed tomography (CT) incidentally revealed a mass in the right upper lung field and lymphadenopathy in the mediastinum and right hilar region (Figure 2), raising suspicion of a malignant lung tumor. Blood tests showed a mildly elevated white blood cell count (WBC: 9400/ μL), without other significant abnormalities. The cerebrospinal fluid (CSF) test revealed a mild elevation in the white blood cell count (Table 1). Based on the clinical presentation, autoimmune encephalitis was suspected, and testing for anti‐neuronal antibodies, including anti‐NMDAR antibodies, was initiated. The case already met the diagnostic criteria for “possible autoimmune encephalitis” proposed by Graus et al. in 2016. Specifically, he fulfilled the following three criteria: [1] acute onset of memory impairment and psychiatric symptoms, [2] CSF pleocytosis (white blood cell count > 5/μL), and [3] reasonable exclusion of alternative causes. These findings strongly supported the diagnosis even before the antibody test results were available. High‐dose intravenous corticosteroid therapy (1000 mg/day for 3 days) was promptly initiated on hospital day 1 in consultation with neurologists, followed by a second course from days 8 to 10. As only partial improvement in neuropsychiatric symptoms was observed after the initial course, the second course was administered to enhance the therapeutic response. On hospital day 12, the corticosteroid therapy was switched to oral methylprednisolone at a dose of 30 mg/day, with gradual tapering. At the time of discharge on hospital day 30, the patient was maintained on 25 mg/day. No clear side effects of the corticosteroids were observed during hospitalization. No psychotropic medications were administered during the clinical course. On hospital day 3, pressured speech and agitation persisted, although an improvement in impaired orientation and short‐term memory impairment was observed based on regular neurological and psychiatric assessments conducted by neurologists and psychiatrists. Pressured speech and agitation subsided by hospital day 8, and emotional stability gradually improved. On hospital day 14, no abnormal behavior was observed. On the same day, anti‐NMDAR antibodies were confirmed in the CSF. Subsequently, bronchoscopy biopsy of the pulmonary mass performed on hospital day 24 confirmed SCLC (Figure 3), leading to a definitive diagnosis of anti‐NMDAR encephalitis associated with SCLC. He recovered without significant neurological sequelae and was discharged in a stable condition on hospital day 30. Regarding oncological management, no chemotherapy was administered during the initial hospitalization. After discharge, he was readmitted under oncologic care to initiate chemotherapy with carboplatin plus paclitaxel for SCLC and has had no recurrence of neuropsychiatric symptoms since then.

**TABLE 1:** CSF test results at admission on the first day of hospitalization.

Parameter	Patient value	Reference range
Opening pressure	120 mmH_2_O	70–180 mmH_2_O
Appearance	Clear and colorless	—
White blood cell count	10/μL	0–5 /μL
Differential count	90% mononuclear cells, 10% polymorphonuclear cells	
Protein	44 mg/dL	15–45 mg/dL
Glucose	81 mg/dL	50–75 mg/dL
IgG	6 mg/dL	1–4 mg/dL
Anti‐NMDA receptor antibody	Positive	Negative

Abbreviation: CSF, cerebrospinal fluid.

## 3 Discussion

This case report describes an 80‐year‐old male with no prior psychiatric history who presented with acute‐onset neuropsychiatric symptoms, including pressured speech, agitation, short‐term memory impairment, and abnormal behavior. In general, anti‐NMDAR encephalitis predominantly affects young females, with an extremely low rate of tumor association in males and a lower incidence of associated tumors in elderly individuals than in younger individuals [7, 8, 13, 14]. This case represents a rare example of anti‐NMDAR encephalitis associated with SCLC in an elderly male. Only a few similar cases have been reported to date [17]. The clinical course is acute, with initial symptoms primarily consisting of psychiatric symptoms such as hallucinations, delusions, anxiety, agitation, and emotional instability, followed by seizures, involuntary movements, autonomic dysfunction, rapid cognitive decline, and impaired consciousness [5, 7, 9]. In contrast, anti‐NMDAR encephalitis in elderly individuals often presents with atypical clinical features compared to younger individuals. Seizures and involuntary movements (such as oral‐lingual dyskinesia) are less likely to occur, whereas psychiatric symptoms and cognitive decline are more commonly observed [11, 12, 13]. Because of this, acute‐onset neuropsychiatric symptoms in older adults are often misattributed to age‐related dementia, delirium, or primary psychiatric disorders, leading to delays in diagnosis. Anti‐NMDAR encephalitis in elderly individuals is particularly challenging to diagnose due to its atypical presentation, lower tumor association rate, and nonspecific imaging findings. In this case, an 80‐year‐old male with no psychiatric history initially presented with various neuropsychiatric symptoms. The symptoms had an acute onset and were accompanied by flu‐like symptoms, leading to the suspicion of autoimmune encephalitis. This suspicion was further supported by a mild elevation in white blood cell count in the CSF test and the presence of a mass in the right upper lung field detected on whole‐body CT on the first day of hospitalization. Following further collaboration with the Internal Medicine Department and completion of antibody testing, the patient was diagnosed with anti‐NMDAR encephalitis associated with SCLC. The neuropsychiatric symptoms were consistent with paraneoplastic encephalitis, likely associated with the underlying malignancy. This case highlights the importance of tumor screening, especially in individuals, and is therefore considered noteworthy. Treatment includes first‐line immunotherapy (high‐dose intravenous corticosteroid pulse therapy, intravenous immunoglobulin administration, and therapeutic plasma exchange), followed by second‐line treatments such as rituximab or cyclophosphamide in refractory cases [18]. In addition, the treatment of malignant tumors is crucial. This is because malignant tumors often trigger an autoimmune response, and treatment of malignant tumors can significantly improve symptoms and significantly contribute to improved prognosis [19, 20, 21]. Anti‐NMDAR encephalitis can sometimes have a severe course, and early diagnosis and treatment are critical factors in determining prognosis [21]. In particular, elderly individuals may have prolonged hospitalization, long‐term cognitive decline, and increased mortality due to poor response to immunotherapy compared to younger individuals [13, 22, 23]. Previous reports involving elderly individuals with SCLC have typically demonstrated limited responsiveness to immunotherapy and poor clinical outcomes [15, 16]. In this case, we considered the possibility of autoimmune encephalitis based on the clinical presentation and imaging findings, and immunotherapy was initiated promptly. As a result, rapid improvement in neuropsychiatric symptoms and stabilization of his physical condition were achieved, enabling early discharge without any serious sequelae. To further contextualize the present case, we reviewed previous reports of anti‐NMDAR encephalitis in elderly patients with malignancies (Table 2) [15, 16, 17, 22]. While anti‐NMDAR encephalitis is a potentially treatable condition, poor outcomes have been observed in a notable proportion of cases. In contrast, a small number of patients—including the present case—achieved early clinical improvement and were discharged without any serious sequelae. These findings emphasize the importance of early diagnosis and timely initiation of immunotherapy, which may result in a favorable clinical outcome. However, given the limited number of case reports, the findings may not be generalizable, and further studies are needed to better understand the clinical characteristics and treatment responsiveness of anti‐NMDAR encephalitis associated with SCLC in elderly patients. This highlights the importance of early diagnosis and treatment of anti‐NMDAR encephalitis to improve clinical outcomes.

**TABLE 2:** Anti‐NMDAR encephalitis in elderly patients (≥ 60 years) with malignancies: Summary of reported cases.

Author	Sex	Age	Underlying disease	Initial symptoms	Treatment for encephalitis	Cancer treatment	Outcome
Kobayashi et al. [15]	M	61	SCLC	Decreased consciousness, Generalized convulsive seizures	IVMP + IVIG	— (diagnosed postmortem)	Death at 1 year
Itagaki et al. [16]	M	66	SCLC	Fever, impaired consciousness (abnormal behavior and disorientation)	IVMP + IVIG + RTX	— (Not feasible)	Death at day 101
García‐Ull et al. [22]	F	74	SCNEC of the pancreas	Behavioral alterations, psychomotor agitation, stupor	IVIG + RTX	Chemotherapy and radiation therapy	Death at 18 months
Sohal et al. [17]	M	72	SCLC	Short‐term Memory loss, anxiety	—	Chemotherapy initiated day 4, Radiation therapy	Improved, survived
Present case	M	80	SCLC	Flu‐like symptoms, pressured speech, agitation, memory impairment, and abnormal behavior	IVMP	Discharged; chemotherapy and radiation therapy initiated post‐discharge	Improved, discharged

Abbreviations: IVIG, intravenous immunoglobulin; IVMP, intravenous methylprednisolone; RTX, rituximab; SCLC, small cell lung cancer; SCNEC, small‐cell neuroendocrine carcinoma.

## 4 Conclusion

This was a rare and clinically significant case of anti‐NMDAR encephalitis in an elderly male with SCLC. Previous reports have described poor responses to immunotherapy and unfavorable prognoses in patients with anti‐NMDAR encephalitis complicated by SCLC. In the present case, early diagnosis and timely initiation of immunotherapy resulted in a favorable clinical outcome. In elderly patients with acute neuropsychiatric symptoms, it is essential to consider autoimmune encephalitis and promptly initiate a diagnostic evaluation and therapeutic intervention, including tumor screening. This case highlights the importance of clinical vigilance and early intervention in anti‐NMDAR encephalitis associated with SCLC.

## Author Contributions

Kohei Kamikawa, Ryohei Takada, Yuya Honda, and Harue Goto treated the patient at our hospital. Kohei Kamikawa, Ryohei Takada, Yuya Honda, and Harue Goto treated the patient in the outpatient clinic. Kohei Kamikawa, Ryohei Takada, Yuya Honda, Harue Goto, and Takashi Okada wrote the manuscript. All authors participated in the discussion, writing, and revisions, and read and approved the final version of the manuscript.

## Ethics Statement

The authors have nothing to report.

## Consent

The patient provided verbal informed consent, and documentation was made regarding the explanation of the method and content, as well as the details of the consent obtained.

## Conflicts of Interest

The authors declare no conflicts of interest.

## Data Availability

All data supporting the findings of this case report are included in the main text of the article. No additional datasets were generated or analyzed during the preparation of this report.
